# The Effectiveness of eHMI Displays on Pedestrian–Autonomous Vehicle Interaction in Mixed-Traffic Environments

**DOI:** 10.3390/s24155018

**Published:** 2024-08-02

**Authors:** Ali Alhawiti, Valerian Kwigizile, Jun-Seok Oh, Zachary D. Asher, Obaidullah Hakimi, Saad Aljohani, Sherif Ayantayo

**Affiliations:** 1 Civil Engineering Department, Faculty of Engineering, University of Tabuk, Tabuk 71491, Saudi Arabia; 2 Department of Civil and Construction Engineering, Western Michigan University, Kalamazoo, MI 49008, USAobaidullah.hakimi@wmich.edu (O.H.); 3 Department of Mechanical and Aerospace Engineering, Western Michigan University, Kalamazoo, MI 49008, USA; 4 Department of Electrical and Computer Engineering, Western Michigan University, Kalamazoo, MI 49008, USA

**Keywords:** external human–machine interfaces, autonomous vehicles, pedestrian–AV interaction, pedestrian safety, mixed-methods approach

## Abstract

External human–machine interfaces (eHMIs) serve as communication bridges between autonomous vehicles (AVs) and road users, ensuring that vehicles convey information clearly to those around them. While their potential has been explored in one-to-one contexts, the effectiveness of eHMIs in complex, real-world scenarios with multiple pedestrians remains relatively unexplored. Addressing this gap, our study provides an in-depth evaluation of how various eHMI displays affect pedestrian behavior. The research aimed to identify eHMI configurations that most effectively convey an AV’s information, thereby enhancing pedestrian safety. Incorporating a mixed-methods approach, our study combined controlled outdoor experiments, involving 31 participants initially and 14 in a follow-up session, supplemented by an intercept survey involving 171 additional individuals. The participants were exposed to various eHMI displays in crossing scenarios to measure their impact on pedestrian perception and crossing behavior. Our findings reveal that the integration of a flashing green LED, robotic sign, and countdown timer constitutes the most effective eHMI display. This configuration notably increased pedestrians’ willingness to cross and decreased their response times, indicating a strong preference and enhanced concept understanding. These findings lay the groundwork for future developments in AV technology and traffic safety, potentially guiding policymakers and manufacturers in creating safer urban environments.

## 1. Introduction

In recent years, the transportation sector has witnessed significant technological advancements, notably the development of AVs. These vehicles are expected to improve traffic safety by mitigating accidents caused by human errors [1,2]. However, their integration into urban environments presents multifaceted challenges, particularly concerning human–AV interactions. Traditionally, the interaction between pedestrians and human-driven vehicles is facilitated through implicit and explicit communication cues such as eye contact, hand gestures, and the flashing indicators of a turning vehicle. Non-verbal signals, such as eye contact, let drivers and pedestrians establish a mutual understanding of when it is safe for pedestrians to cross the street [3]. These communication methods are particularly important in locations without clear regulation such as unmarked crossings [4]. However, with the introduction of AVs, the conventional, human-centric cues recede, necessitating innovative solutions to bridge the emergent communication gap.

Recently, the researchers, manufacturers, and designers of AVs have been focusing on this issue by proposing external human–machine interfaces (eHMIs) as interaction features between autonomous vehicles and other road users to ensure safety [4,5,6,7,8,9,10]. These interfaces are integrated into the vehicle or traffic environment to provide information to road users about the AV’s movement intentions, its mode of operation, awareness of the surroundings, and other information [11]. To date, more than 70 eHMI concepts have been proposed, with most found to be beneficial for road users [12]. The existing eHMI evaluations have predominantly been conducted in laboratories, utilizing virtual reality (VR) and monitor-based simulations, focusing on simple one-to-one traffic scenarios, such as a single pedestrian interacting with a single vehicle [13,14,15]. 

Research indicates a significant gap in understanding how eHMIs can effectively convey messages to multiple users at once [16,17]. As a result, there is a pressing need to evaluate eHMI concepts in scenarios involving multiple road users in real-world settings. This issue is compounded by the lack of agreement in the research community regarding the specific types of information that eHMIs should convey [18]. Predominantly, eHMI concepts have centered on signaling the vehicle’s intention to yield, leaving other crucial communication information less explored [12]. 

Additionally, there is an open question about the effectiveness of eHMI designs over time. As pedestrians repeatedly interact with AVs and become familiar with their communication methods, it is unclear if their initial perceptions will remain unchanged or evolve over time. This aspect is vital for the continued development of eHMI technologies to ensure they remain effective as user familiarity grows and traffic scenarios become more complex. These issues suggest that many eHMI aspects are not fully addressed by previous research. 

To fill these gaps, we employed a mixed-method approach, integrating controlled experiments with a broad survey study This study aims to evaluate the effectiveness of various eHMI configurations on pedestrian behavior and safety in mixed-traffic scenarios. We hypothesize that certain eHMI designs will significantly improve pedestrians’ understanding of AV intentions, thereby enhancing their willingness to cross and reducing response times. Our study centered around two outdoor experiments using an auto-pod shuttle, involving 31 participants in the initial experiment and 14 in a follow-up session. Additionally, we conducted an intercept survey, further enriching our insights with data from 171 individuals. We employed a diverse range of eHMI designs, from simple flashing green LEDs to more complex displays featuring robotic signs and countdown timers, and compared these against a control scenario with no eHMI. Our comprehensive approach encompassed both one-to-one and multi-user interactions to reflect the diverse conditions of urban traffic environments. By analyzing the participants’ willingness to cross, crossing response times, and feedback on safety and display information clarity, our study aims to guide the creation of scalable and effective eHMIs, ultimately enhancing road users’ safety.

This study aligns with the aim and scope of the Sensors journal by focusing on the development and evaluation of eHMIs for AVs, which are critical devices/sensors designed to convey AV information to other road users. By addressing the real-world applicability of eHMIs in complex traffic scenarios, our research contributes to the field of human–machine interaction, a key area within the scope of the Sensors journal.

## 2. Related Work

### 2.1. Pedestrian–AV Communication

The rapid evolution of AVs presents new challenges in the realm of road safety, particularly concerning interactions with vulnerable road users like pedestrians and cyclists. The literature underscores the critical importance of transparent communication between AVs and pedestrians [19]. In the era of fully autonomous vehicles where there is no human driver, the traditional forms of communication between humans, such as eye contact or hand gestures, must be substituted with new approaches. This is crucial to pedestrians to guarantee a clear understanding of AVs’ intentions and actions, thereby enhancing safety [20,21,22]. External HMIs have emerged as a pivotal tool in mediating these interactions [7]. Utilizing eHMIs in AVs has been shown to promote early pedestrian crossings, enhance the feelings of safety and confidence, improve the overall crossing experience, and increase trust in AVs [23,24,25].

More than 70 eHMI concepts have been proposed to facilitate the interaction between AVs and road users [12]. The existing and proposed eHMI technologies involve both auditory and visual displays built into the vehicle, signals sent by the AV to the pedestrian via a smartwatch or other device carried by the pedestrian, and displays situated in the traffic environment, such as displays set into or projected onto the roadway [4,5,6,10,26]. These interfaces are designed to convey different types of information, like AVs’ driving mode (autonomous/manual), intention (yielding/not yielding), awareness of the surroundings, advice, and other information [27,28,29,30]. While there remains much more investigation to be carried out in determining the most effective and safe eHMI technologies, almost all the research to date finds that vehicles equipped with eHMI are perceived as more reliable, safe, and effective than interacting with AV not equipped with eHMI features [31].

### 2.2. Information Types of eHMIs

Investigators have examined types of eHMI information content. According to [30], these types are divided into four groups: vehicle’s driving mode, vehicle’s intention, giving advice, and perception of the surroundings. Previous research has not reached a consensus on the most effective type of information that eHMIs should display [18]. For example, while some studies found that giving advice or perception information is more important than showing a car’s intention [4,29], other researchers indicated that road users should not be advised by eHMIs [9,32]. Other studies, such as [6,33], found no difference in pedestrian behavior between the two modes of content delivery. Moreover, the authors of [34] investigated how pedestrians felt and reacted around AVs without eHMI, comparing them to AVs with eHMI displaying vehicle status, intention, or both. The results showed that participants felt safer and more trusting when an AV had some form of eHMI and also that the highest ratings were given to AVs displaying both status and intent which made participants feel the safest. Other information such as showing vehicle speed on the display was used in previous research. A study by [6] highlighted the comparative efficacy of different communication modes: explicit advice and vehicular speed information. Interestingly, no substantial difference was observed between these modalities. Furthermore, given these divergent findings, there is a pressing need for further research on this topic, ensuring that autonomous vehicles can communicate effectively and safely with all road users.

### 2.3. Scalability of eHMIs

Scalability, in the context of eHMI, refers to the interface’s ability to convey unambiguous messages to multiple road users simultaneously [14]. According to [35], eHMI may lead to misinterpretations in ambiguous traffic situations if it is not well designed. The authors of [34] discuss the need for eHMI to be easily perceived under a wide variety of environmental conditions without creating distraction and that this technology must be “intelligible, unambiguous, and scalable” to adapt it to multiple, simultaneous vehicles and pedestrians. The ability of eHMIs to convey clear messages to different road users at once is an essential criterion for effective design, ensuring safety and preventing confusion in complex traffic scenarios. The scalability challenge, essential in multi-vehicle and multi-pedestrian scenarios, has been a focal point of recent investigations [13].

While eHMIs have the potential to improve safety and efficiency on the road, the majority of the current concepts are designed only for one-to-one interaction, where the AV interacts with a single road user at a time [13,14,20]. This approach fails to take into account the complex interactions that occur on the road, where multiple road users interact with each other simultaneously. In such situations, different road users may perceive the eHMI differently, leading to misunderstandings and even dangerous circumstances [14]. For instance, if an AV decelerates to yield to a specific pedestrian at a crosswalk, other pedestrians might incorrectly assume the vehicle is slowing down for them. Research on the ability of eHMI concepts to clearly convey messages to multiple road users is limited, with only a few studies investigating their scalability [15,16]. For example, in their study, the authors of [14] utilized virtual reality to investigate pedestrian interactions with AVs, assessing various eHMIs and their effectiveness beyond one-to-one interactions. Their findings suggest that while road projections can clarify an AV’s intentions, this may not be universally applicable. Similarly, using an online video study, the authors of [35] found that a flashing LED interface was more likely to encourage pedestrian crossing compared to a static LED, particularly when another pedestrian was present. Out of the 54 papers reviewed by [15], only 11 delved into the complex dynamics of multi-pedestrian scenarios, highlighting a research gap in scenarios involving multiple pedestrians.

### 2.4. Evaluation Methods of eHMIs

The evaluation of eHMI designs has been approached through various methods, each with its own strengths and challenges in measuring the clarity and effectiveness of the signals communicated to road users. Online surveys, such as those conducted by [5,36,37], have presented participants with different eHMI concepts, typically asking them to assess the safety of crossing based on the eHMI displayed. While surveys can involve a high number of repetitions and variations, they rely on participants’ imaginations to predict their actions, which can limit the validity of the findings [38]. Moreover, lab-based setups and VR environments have also been utilized to immerse participants in traffic scenarios, offering a more interactive experience. For instance, Refs. [4,7] employed head-mounted displays (HMDs) to simulate eHMI-equipped AVs, with results indicating that eHMIs significantly increased perceived safety. HMDs, by providing a wider visual field, have the advantage of simulating a more realistic interaction with AVs than static images or videos used in surveys. Another evaluation method is field tests on closed roads, such as those by [6,39], which allow participants to engage with actual vehicles, providing a more naturalistic setting. However, these studies often restrict participants’ actual crossing due to safety and ethical considerations. 

The authors of [15] conducted a comprehensive analysis of the research landscape concerning the scalability of eHMIs, focusing on their utility in complex traffic situations involving multiple vehicles or pedestrians. In their extensive review of 54 papers, they highlighted the prevailing research gap in the empirical examination of eHMIs in real-world environments. Among the studies they analyzed, only a single paper ventured to test eHMI concepts on public roads. This solitary study underscores the rarity of research conducted outside controlled or simulated environments and points to a significant need for more field-based research to validate eHMI effectiveness in real-world multi-road user scenarios.

## 3. Methods

Our study was conducted through a combination of two experiments and an intercept survey. We conducted real outdoor experiments where pedestrians interacted with an auto-pod shuttle representing an AV. Our experimental design was methodically divided into two experiments, initial and follow-up. A total of 31 participants were initially recruited for the study, ensuring a diverse representation across age groups and genders. A subset, 14 participants, from the initial study agreed to return after a three-week interval for the follow-up experiment. The participants were recruited through flyers distributed across various locations. The participants signaled their interest by scanning a QR code directing them to a Qualtrics questionnaire where they provided demographic details. They were recruited based on a diverse range of demographics, including age, gender, and previous experience with AVs. The inclusion criteria included individuals aged 18 and above, with a balanced representation of genders. Exclusion criteria were applied to ensure safety and ethical considerations, such as excluding individuals with mobility and visual impairments that might affect their ability to participate in the study. All the participants provided informed consent before participation. The participants were compensated $20 for their involvement in each session. 

In parallel, to broaden our understanding of pedestrian perspectives on AV interactions, we carried out an intercept survey, engaging an additional 171 participants. This survey, taking about 5 min to complete, sought the participants’ responses to different eHMI displays, similar to the ones used in the experiments. The study methods and protocols were approved by the Western Michigan University Human Subjects Institutional Review Board.

### 3.1. Experiments 

#### 3.1.1. Location and Apparatus

**Figure 1:**
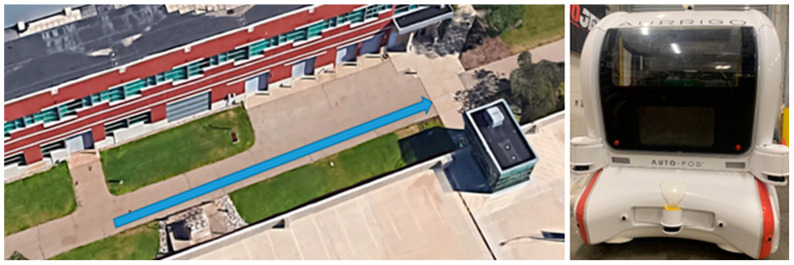
Experiments’ location (**left**) and automated shuttle used for eHMI testing (**right**). The blue arrow shows the AV path.

Both experiments took place on an outdoor street of the Engineering College at Western Michigan University (see Figure 1, left). This particular street, located between a parking lot and the college’s offices and laboratories, represents a high-traffic pedestrian crossing area. This location choice was pivotal in replicating the typical conditions of an urban crossing environment, adding valuable realism to our study. The vehicle used in the study was the Aurrigo Auto-pod, a 4-seater low-speed (up to 15 mph) automated (SAE Level-4) shuttle [40] (Figure 1, right). The auto-pod is equipped with advanced sensors and a 24″ monitor, making it an ideal candidate for testing eHMIs for pedestrian–AV interactions. To not distract the participants, the built-in LED lighting on the exterior of the vehicle was removed. 

#### 3.1.2. Evaluated eHMI Displays

**Figure 2:**
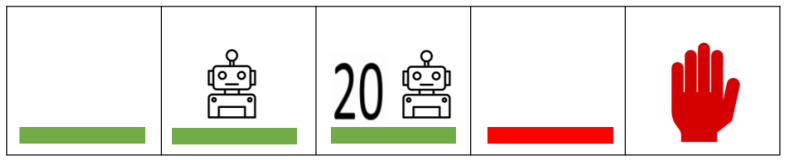
Interfaces used in both experiments.

The eHMI designs in this study were designed by the participants in our previous discussion groups and drawing sessions research [41]. The concepts (Figure 2) under investigation were as follows:

- Flashing Green LED

The flashing green LED display was selected as one of the basic visual cues to represent a standard, universally understood signal. Green, in traffic, typically conveys safety or permission. The flashing attribute was incorporated to catch the pedestrian’s attention more effectively than a static light might [35]. It serves as a foundational eHMI condition against which more complex displays can be compared.

- Flashing Green LED combined with Robotic sign

Building on the basic green LED, this design integrates a robotic sign. The intention behind combining the green LED with a robotic sign is to bridge the familiarity of a traditional traffic signal with the futuristic aspect of autonomous vehicles. The robotic sign acts as a symbol of automation, ensuring pedestrians recognize that they are interacting with an autonomous entity.

- Flashing Green LED, Robotic sign, and Countdown Timer

This eHMI design has more information. Along with the green LED and robotic sign, it introduces a timer, providing pedestrians with a dynamic countdown timer. The inclusion of a timer offers an additional layer of information, letting pedestrians know how much time they have to cross safely before the vehicle starts moving.

- No eHMI

The absence of any eHMI display serves as a control condition in our study. It represents the scenario of encountering an autonomous vehicle without any specialized signaling. By comparing results from this condition to others, we can gauge the effectiveness and necessity of introducing eHMI displays for pedestrian safety and comfort.

- Flashing Red LED

The flashing red LED design was thus chosen to investigate the dichotomy of interpretations surrounding the color. By analyzing pedestrian reactions to the flashing red LED, we aim to unpack whether the conventional warning symbol holds or if it is perceived as an unexpected green light in the context of autonomous vehicles.

- Red Hand

The upraised red hand is a universally recognized traffic signal indicating “do not cross” or “stop” In the context of our experiment, this symbol was employed to represent an autonomous vehicle that will not stop. Introducing this symbol was crucial to counteract any assumptions from the participants who might expect the AV to always stop for them.

We did not select any display that contained advice information like “walk” because they were found to be problematic and may put road users in a dangerous situation [18].

#### 3.1.3. Experimental Design and Procedure

**Figure 3:**
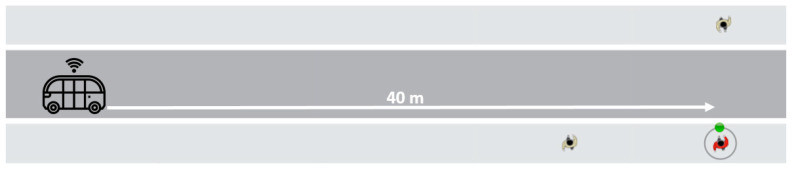
A top view of the experiment environment. The circled red pedestrian represents the participant.

The study was designed to explore interactions between the participants and an AV equipped with various eHMI displays in different traffic scenarios. In the initial study phase, the participants were exposed to two road scenarios:

1. Scenario 1: One-to-one interaction, an interaction solely between the pedestrian and the AV. This was foundational for understanding pedestrian perceptions and reactions without the influence of other road users.
2. Scenario 2: One-to-multi-interaction, the pedestrian interacted with the AV in the presence of other road users (two pedestrians), replicating the complexities of real-world urban environments (Figure 3).

**Figure 4:**
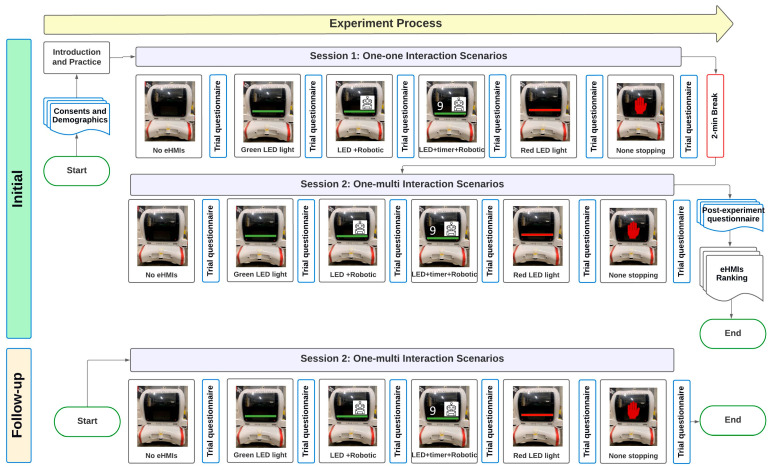
Experiments’ process. The initial experiment (**upper**) has two sessions where the participants interact with the AV only or with the AV and the other pedestrians. In the follow-up sessions (**lower**), the participants interact with the AV and multi-pedestrian scenarios only.

Upon arrival at the experiment location, the participants were oriented to the experimental setup. Before starting the primary trials, a practice session was conducted to familiarize the participants with the testing procedure (Figure 4). 

**Figure 5:**
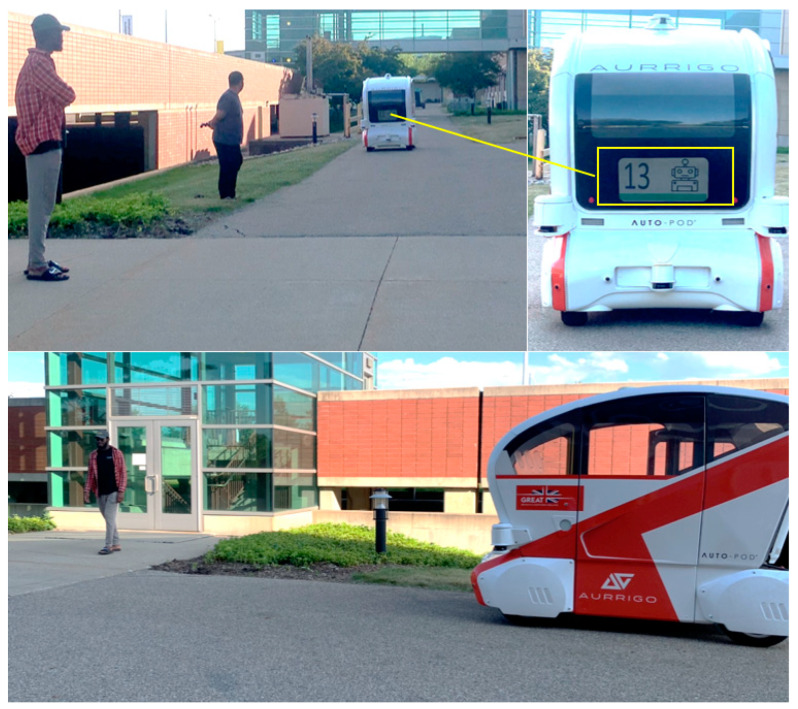
Photographs showing the interaction of the pedestrians with the AV.

The AV initiated its approach from a distance of 50 m away from the participant. The participants, stationed at the curbside, were instructed to observe the approaching AV (Figure 5). At a point approximately 40 m away, the AV began displaying its eHMI cues, maintaining a controlled speed of about 4 mph. This speed was chosen for safety considerations and to give the participants enough time to process the eHMI signals. The participants were tasked to focus on these cues and determine their crossing decision as soon as they felt safe. To ensure participant safety, crossing the street was simulated by raising a hand instead of actual crossing. This gesture served as a non-verbal communication of their decision, ensuring safety, and minimizing potential risks. The practice session concluded with the AV continuing its approach and coming to a complete stop about 2–1.5 m away from the participant. The auto-pod’s movements were controlled remotely to ensure participant safety. The participants were informed that the AV might or might not stop.

Post the practice interaction, the participants entered the primary trials, where they navigated through two distinct road scenarios, each featuring AVs equipped with various eHMI displays. In total, each participant had 12 trials (2 scenarios × 6 displays). They were continuously reminded that the AV’s decision to stop or continue would vary across scenarios, reinforcing the unpredictability factor. After each trial, the participants provided their ratings on specific metrics, capturing their perceptions, feelings, and interpretations of the eHMI displays. After the first six trials, they had a few minutes of break. Concluding the primary trials, the participants filled out a post-experiment questionnaire. The experiment took about one hour for each participant.

A subset of the initial participants was re-engaged for a follow-up study. The 14 participants for the follow-up were the only ones who agreed to return from the initial pool of 31 participants. This subsequent study closely mirrored the procedures of the initial experiment but was specifically focused on the second road scenario. One of the primary objectives of our research was to gain a deeper understanding of pedestrian perceptions of eHMIs, especially in mixed-traffic situations. Given the extensive nature of the initial study, which could take up to an hour, it was logistically challenging to cover both scenarios comprehensively in one session without risking participant fatigue or reduced attention. Moreover, we were keenly aware of the importance of retaining participant willingness for a return engagement. By focusing the follow-up study on just the second road scenario, we ensured a more manageable time commitment for our participants, increasing the likelihood of their return and ensuring the quality of their feedback.

#### 3.1.4. Data Collection and Analysis

Various questionnaires were employed at different stages of the experiment. Pre-experiment questionnaire: gathered information on demographics, the frequency of road crossings, familiarity with AVs, and general thoughts about AVs. Post-scenario questionnaire: assessed participant perceptions after each scenario using a Likert scale (1–7). Post-experiment questionnaire: Using open-ended questions, the participants were asked about the type of information they wanted to see, challenges or difficulties they faced during their engagements with the AV, the interpretations of the used displays (green LED light, red LED light, countdown timer, and robotic sign), and suggestions to improve the eHMI designs or their general interaction experience. Lastly, they were asked to rank the eHMI designs. 

Multiple analysis methods were used in this research including descriptive statistics, Repeated Measures ANOVA (RM ANOVA), paired *t*-tests, mixed-effects model, and thematic content analysis.

#### 3.1.5. Measures

We assessed multiple metrics to gain a comprehensive understanding of the participants’ interactions and perceptions regarding the eHMI displays. One of the primary measures was the “willingness to cross”. This metric gauged the participants’ confidence in crossing the road based on the AV’s display signals. The participants were asked to raise a hand to indicate their willingness to cross, with the act of raising the hand being recorded as a crossing indication. Furthermore, we quantified the “response time to cross”. This metric captured the participants’ response time (in seconds) in making crossing decisions. Specifically, the response time was defined as the interval from the moment a participant first noticed the approaching AV to the instant they indicated their intent to cross by raising their hand. The moment a participant first noticed the approaching AV was identified by observing when the participant turned their head and looked directly at the AV. This was carefully noted by researchers and emphasized during the training session to ensure accuracy.

After each interaction scenario, the participants rated multiple metrics on a scale of 1–7 provided in the post-scenario questionnaire. We evaluated “safety feelings”, providing insight into how secure the participants felt when making crossing decisions when interacting with the AV. Another critical measure was the “understanding of the eHMI messages”. This metric was collected from rating questions on the ease of understanding the display. In addition, “addressing perception” was measured to know how directly they felt the display’s message was addressing them. This measured their sense of engagement and personal relevance with the AV’s communication. Being “well informed” is another metric we used in this study. The participants indicated the extent to which they felt informed by the eHMIs on the vehicle. The effectiveness of eHMIs has been measured by subjective and objective indicators such as perceived safety, willingness to cross, trust, crossing speed, and crossing response time [5,33,38,42].

### 3.2. Intercept Survey 

As part of our comprehensive research on pedestrian interactions with AVs and the effectiveness of eHMIs, we conducted an intercept survey that complemented our outdoor experiments. This survey was instrumental in expanding our understanding by capturing a broader range of perspectives on pedestrian perceptions of AV interactions. The survey successfully engaged a total of 171 participants. These individuals were recruited from a variety of locations to ensure a diverse representation across different demographics. The survey was designed to be concise yet comprehensive, taking approximately 5 min to complete. 

After collecting demographic information, the participants were shown a picture to help them visualize interacting with an approaching AV equipped with eHMIs in a mixed-traffic environment. This setup was crucial for aligning their mindset with the scenario of interest. Four different eHMI displays were used: a flashing green LED light, a flashing red LED light, a combination of flashing green LED light and a robotic sign, and a combination of flashing green LED light, a robotic sign, and a countdown timer. The participants were then asked to express their willingness to cross the road in front of the AV for each displayed eHMI. Additionally, they evaluated their understanding of each eHMI using a Likert scale, focusing on aspects like the AV’s intention to stop, its automated mode, and the overall ease of understanding the display. Finally, the survey concluded with the participants ranking their preferences for the eHMI displays.

## 4. Results

### 4.1. Initial Experiment

#### 4.1.1. Demographic Data

The study’s participant demographic was diverse, encompassing a range of ages and attitudes toward AVs. Of the participants, 55% identified as male, while 45% were female, presenting a balanced gender distribution. In terms of age groups, 42% were between the ages of 18 and 24. This was closely followed by the 25 to 40 age group, representing 36% of the participants. Those aged 41 to 64 made up 16%, while a smaller proportion, 6%, were above the age of 64. The participants’ previous experience with autonomous vehicles varied. A minority, 16%, reported having prior interaction with an AV, suggesting that for the majority (84%), this study was among their first experiences with such technology. Regarding their general perceptions of autonomous vehicles, the majority of the participants (61%) held a positive view, 23%, remained neutral, while 16% expressed negative sentiments.

#### 4.1.2. Willingness to Cross 

**Figure 6:**
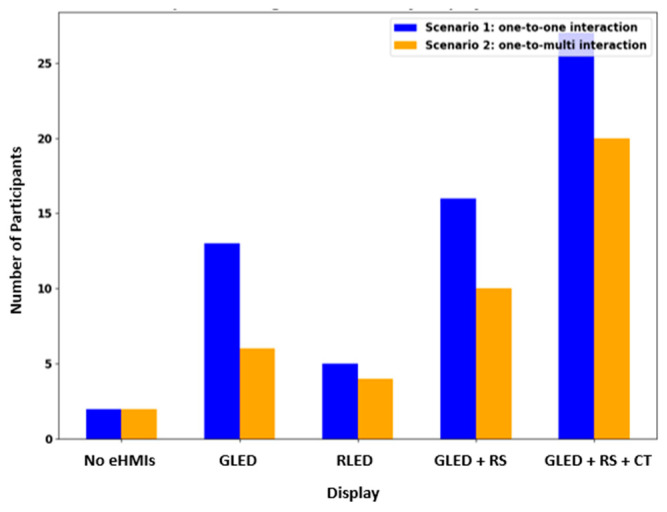
Number of pedestrians who are willing to cross by display and scenario. GLED is green LED; RLED is red LED; GLED + RS is green LED + robotic sign; GLED +RS + CT is green LED + robotic sign + countdown timer.

The willingness of the participants to cross based on the eHMI displays offers insightful trends (see Figure 6). In Scenario 1, about 87.10% of the participants were willing to cross with the green LED, robotic sign, and timer configuration, showcasing its effectiveness in instilling confidence. However, in the absence of any eHMI, a mere 6.45% felt comfortable crossing. The dynamics in Scenario 2 shifted slightly but still echoed similar sentiments. Here, 64.52% of the participants were willing to cross with the green LED combined with the robotic sign and timer. Yet, without any eHMI, only 6.45% were inclined to cross, underlining the critical importance of clear and intuitive eHMI designs in fostering pedestrian trust and confidence.

Statistical analysis using the Chi-Square Test of Independence revealed significant associations. The type of eHMI display showed a significant relationship with the participants’ willingness to cross, with a Chi-Square statistic of 81.95 and a *p*-value less than 0.001. This suggests that the specific eHMI display used plays a crucial role in influencing a participant’s decision to cross the road. Additionally, the scenario itself, whether it was a one-to-one or one-to-multi interaction, also significantly influenced the willingness to cross, as indicated by a Chi-Square statistic of 5.76 and a *p*-value of 0.016. Furthermore, the combined effect (interaction) of the eHMI display type and scenario yielded a Chi-Square statistic of 91.67 and a *p*-value less than 0.001, reaffirming the profound impact of these factors on pedestrian behavior. Collectively, these findings emphasize the pivotal role of eHMI displays and road scenarios in shaping pedestrian decisions and perceptions.

#### 4.1.3. Crossing Response Time

**Figure 7:**
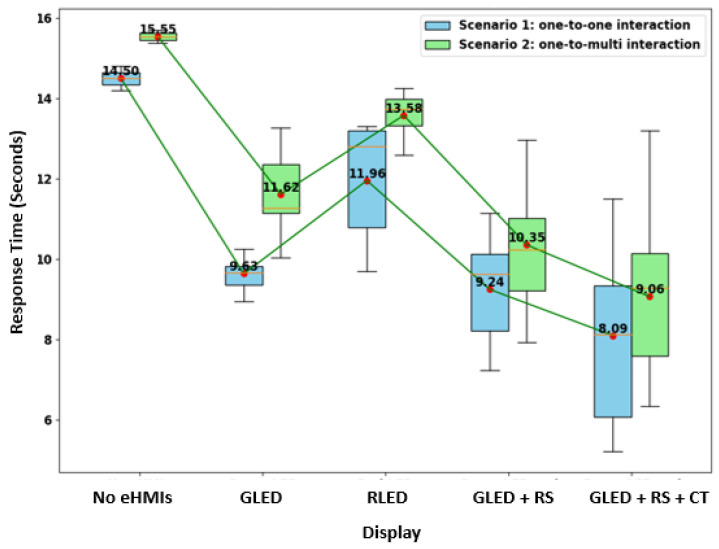
Boxplots of participants’ crossing response times in seconds per display and scenario.

In analyzing the decision times across the various eHMI displays, clear patterns emerge (see Figure 7). For Scenario 1, the display of the green LED combined with the robotic sign and timer resulted in the shortest mean decision time of 8.09 s. In stark contrast, the absence of any eHMI led to the longest decision time, averaging 14.50 s. Scenario 2, which introduced additional complexities with other road users, saw a general increase in decision times across all the eHMIs. Yet, the green LED with the robotic sign and timer remained the most efficient, with the participants taking an average of 9.06 s to decide. 

To analyze the decision time data in the context of different eHMI displays and scenarios, we employed a mixed-effects model (Table 1). This model was chosen due to its robustness in handling repeated measures, such as ours, where each participant interacted with multiple eHMI displays across different scenarios.

The baseline response time when no eHMI was present and under the conditions of Scenario 1 (an interaction solely between pedestrians and the AV) was gauged at approximately 12.329 s. Delving deeper into the display effects, the presence of the green LED was found to lead to a significant decline in the decision time by an average of 3.982 s in contrast to the baseline (*p* = 0.028). Similarly, the display “green LED and robotic sign” culminated in a notable decrease of roughly 4.865 s in the decision time relative to the baseline (*p* = 0.006). The “green LED and robotic sign and timer” display also had a significant bearing, reducing the decision time by around 5.465 s when compared to the baseline (*p* = 0.002). However, the influence of the red LED, which curtailed the decision time by an estimated 2.156 s, was not statistically significant (*p* = 0.272).

When we explored the effect of the scenario, transitioning to Scenario 2, wherein the participants interacted with the AV amidst other road users, the decision time saw an increment by an average of 1.354 s. This effect, intriguingly, did not attain statistical significance (*p* = 0.193). In simpler terms, whether the participants were alone or amidst other road users, their decision time was, on average, consistent. This might mean that the mere presence of other road users does not necessarily influence how quickly the participants decide to cross the road when interacting with the AV. 

Interaction effects explore whether the impact of one factor (e.g., eHMI displays) changes depending on the level of another factor (e.g., scenario). All the interaction terms emerged as non-significant, suggesting that the influence of the eHMI displays on the decision time remains consistent regardless of the scenario. This means that, for example, the effect of the “green LED and robotic sign” display on the decision time is similar in both one-to-one interactions (Scenario 1) and one-to-multi interactions (Scenario 2).

In sum, while specific eHMI displays evidently influence the decision times, the surrounding context (whether the participants are alone or with other road users) does not seem to play a significant role in modifying these effects. This could imply that the clarity, intuitiveness, or relevance of the eHMI displays is paramount, with external factors like the presence of other road users having a minimal bearing on decision-making times.

#### 4.1.4. Subjective Ratings

**Figure 8:**
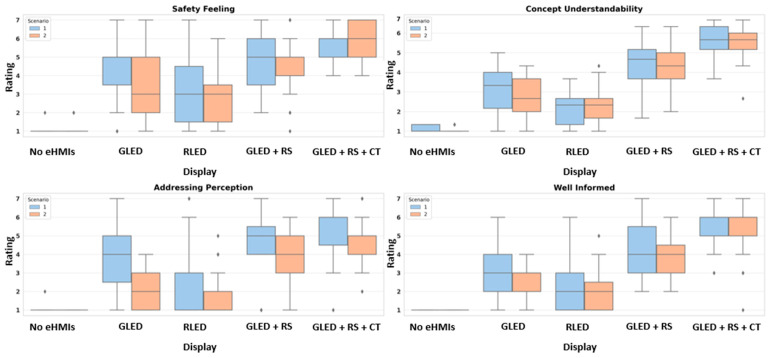
Distribution of participant ratings for safety feeling, concept understandability, addressing perception, and being well informed.

Figure 8 presents the boxplots illustrating the participants’ ratings for the various eHMI displays across the two road scenarios. Each boxplot visually captures the central tendency, spread, and potential outliers for each eHMI display and scenario combination. 

In terms of safety feelings, the participants’ feelings varied distinctly across eHMI displays and scenarios. The RM ANOVA revealed a significant effect of the eHMI display on safety feeling (*F*(4, 120) = 79.46, *p* < 0.0001), the road scenario (*F*(1, 30) = 13.90, *p* = 0.0008), and their interaction (*F*(4, 120) = 3.75, *p* = 0.0065), suggesting that certain displays’ ability in conveying safety might differ depending on whether the pedestrian is interacting solely with the AV or amidst other road users. Descriptively, when there was an absence of any display, the participants’ safety feelings were noticeably lower in both scenarios. The combination of green LED with robotic sign and timer notably received higher ratings, indicating a heightened feeling of safety, especially in Scenario 1. Scenario 2 displayed a marginally reduced average safety feeling as compared to Scenario 1 in this context.

For concept understandability, the RM ANOVA highlighted a significant effect of the eHMI display (*F*(4, 120) = 202.48, *p* < 0.0001) and an interaction effect with the road scenario (*F*(4, 120) = 3.53, *p* = 0.0092). However, the road scenario did not significantly influence this perception (*F*(1, 30) = 2.38, *p* = 0.1333). Figure 8 shows that the displays featuring robotic signs or robotic signs and timers alongside the green LED were most comprehensible to the participants in both scenarios. Both these displays received consistently higher ratings, indicating that the participants found them particularly intuitive and straightforward. Conversely, the “no eHMI” display yielded the lowest ratings.

Regarding the participants’ feelings addressed by the display, the RM ANOVA indicated that there were significant effects of the eHMI display (*F*(4, 120) = 91.54, *p* < 0.0001), the road scenario (*F*(1, 30) = 38.28, *p* < 0.0001), and their interaction (*F*(4, 120) = 8.32, *p* < 0.0001). The participants felt most addressed by the displays when they incorporated a robotic sign, especially when combined with a timer, particularly in Scenario 1. This is supported by the highest mean score of 5.3 in Scenario 1 for the display incorporating both the robotic sign and timer.

Lastly, for the feeling of being well informed by the display, the RM ANOVA showed a significant effect of the eHMI display (*F*(4, 120) = 132.56, *p* < 0.0001) and the road scenario (*F*(1, 30) = 6.73, *p* = 0.0145); however, their interaction was not statistically significant, indicating a more consistent effect of the displays across both scenarios. The participants reported feeling most informed by the eHMI displays that incorporated both a robotic sign and a timer. The absence of any eHMI display resulted in the lowest scores for feeling informed across both scenarios.

#### 4.1.5. Analysis of Post-Experiment Responses

For the post-experiment phase, we conducted a detailed analysis of the participant feedback, breaking it down question by question. Utilizing the MAXQDA software (Release 2020. 4. 2) for thematic content analysis, we first coded the qualitative data from each question separately, identifying predominant sentiments and concepts within the participants’ responses. This approach allowed us to extract specific themes from each question.

Desired Communication Information: Regarding the question of what communication information they would like to receive from the AV, the responses generally united around a few primary themes. The majority (21 participants) highlighted the importance of knowing the AV’s operational mode—whether it is in autonomous mode or not, and information on the AV’s intentions, such as if it is going to stop or continue. Some (seven participants) expressed a preference for straightforward directives such as “walk” or “stop”, rather than relying solely on color-coded symbols which could be open to interpretation. Five participants mentioned the importance of timers as information on the display. Two participants wanted to know the location where the AV will stop.

Interaction Challenges: When discussing challenges faced during their interaction with the AV, a few (three) participants conveyed feelings of uncertainty about eHMI colors, which sometimes led to confusion. Six participants felt that the AV might not be aware of their presence.

Interpretation of Displays: Delving deeper into the meaning attributed to specific displays, the majority (23 participants) perceived the green LED light as an indicator of safety, signaling them to proceed or cross. Conversely, the red LED light was largely seen as a warning or directive to halt, but some mentioned the opposite. A majority of the participants understood the countdown timer as indicating the time left to cross. The robotic sign was more straightforward, with most (26 participants) recognizing it as an indication of the vehicle operating in an autonomous mode.

Suggestions for Improvement: Further suggestions from the participants on potential improvements or additions to the display brought forth some ideas. Eight participants recommended that the inclusion of audible messages or sounds could help those with visual impairments. Four participants suggested using text instructions like “walk” or “stop”. Three participants mentioned the need for brighter displays or larger signs to make the eHMIs more visible from a distance or in bright daylight.

**Figure 9:**
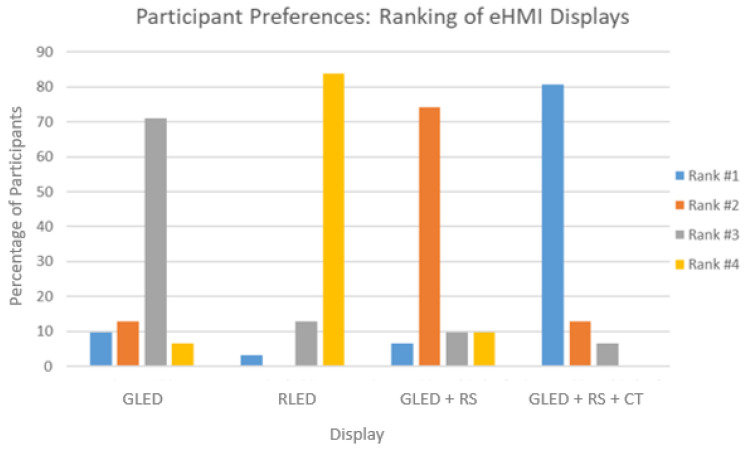
Participants’ ranking for their preferred eHMI concept. Rank #1 represents the best choice and Rank #4 represents the lowest.

Ranking of eHMIs: Lastly, the participants ranked the eHMI designs, which offered a hierarchical understanding of their preferences and showed which designs were most intuitive and effective from a user standpoint (see Figure 9). The eHMI design combining the green LED, robotic sign, and timer emerges as the most preferred, with approximately 81% of the participants ranking it as their top choice. Conversely, the red LED design was the least popular, with roughly 84% of the participants ranking it as their least preferred choice. The green LED combined with the robotic sign experienced a mixed reception; while around 74% of the participants ranked it as their second choice, few had varying opinions, placing it either as their first, third, or fourth preference. The standalone green LED saw a majority, nearly 71%, ranking it as their third choice.

### 4.2. Follow-Up Experiment 

To understand the potential effect of familiarity or repeated exposure to eHMIs on pedestrian behaviors and perceptions, a subset of the participants was invited back for a follow-up session three weeks after their initial participation. During this session, they engaged with the same eHMI displays, but only in the second road scenario. The responses of these 14 participants in the follow-up were then directly compared with their responses from the initial experiment to assess changes and trends.

#### 4.2.1. Demographic Data 

The gender composition was moderately male-dominant, with males accounting for 57% and females for 43% of the participants. Half of the participants, 50%, were in the 18–24 age range, 29% of the participants were 25–40 age group, 41–64 age group accounted for 14% of the participants, and 7% of them were above the age of 64. 

#### 4.2.2. Willingness to Cross and Crossing Response Time

**Figure 10:**
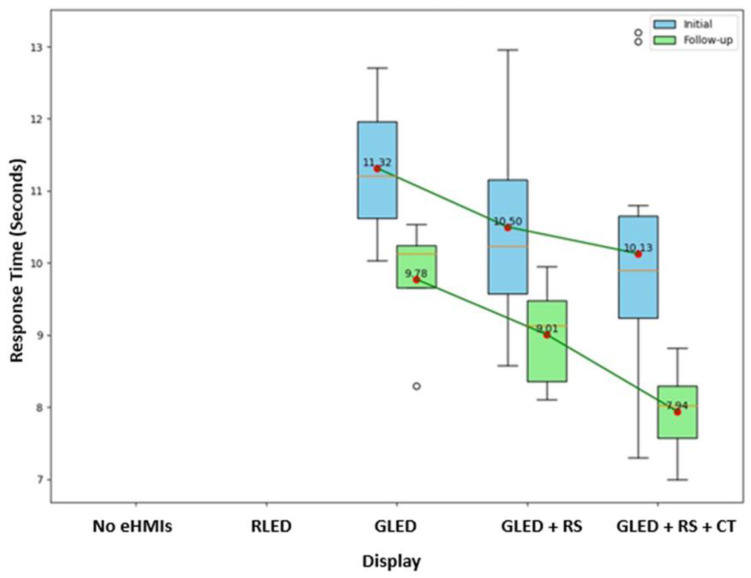
Comparison of participant crossing response time in seconds across displays and sessions.

Table 2 and Figure 10 provide insight into how the participants’ perceptions evolved over the three-week interval, especially when it comes to their willingness and response time to cross in front of the AV based on the eHMI displays.

From Table 2, we can observe that for every eHMI display, there is an increase in the percentage of the participants willing to cross in the follow-up session compared to the initial session. The most pronounced change is seen for the “green LED and robotic sign and timer” display, where the willingness reached 100% in the follow-up session. Moreover, the boxplots in (Figure 10) illustrate a comparative analysis of the participants’ response times across the different eHMI displays in two sessions: initial and follow-up. Notably, the “green LED and robotic sign and timer” consistently displayed the shortest decision times, underscoring its effectiveness in facilitating quick pedestrian decisions. 

To determine if there are significant differences in the participants’ willingness to cross and decision time across the displays, we used paired-sample *t*-tests. In terms of the “willingness to cross”, our analysis revealed significant differences between the initial and follow-up sessions for the “green LED and robotic sign” (*p* = 0.040) and the “green LED and robotic sign and timer” (*p* = 0.032) displays. This signifies that the participants’ willingness to cross in the presence of these eHMIs shifted over the three weeks, possibly indicating a change in their trust or comfort levels with these displays. In addition, for the “response time” metric, a noteworthy finding was associated with the “green LED and robotic sign and timer” display. The participants, on average, took significantly less time to decide in the follow-up session compared to the initial one (*p* = 0.001). This reduced decision time, particularly for the “green LED and robotic sign and timer” eHMI, suggests that familiarity or repeated exposure to certain displays can lead to more efficient or confident decision making by pedestrians when interacting with specific eHMIs. All the other displays were not statistically significant. 

#### 4.2.3. Subjective Ratings

**Figure 11:**
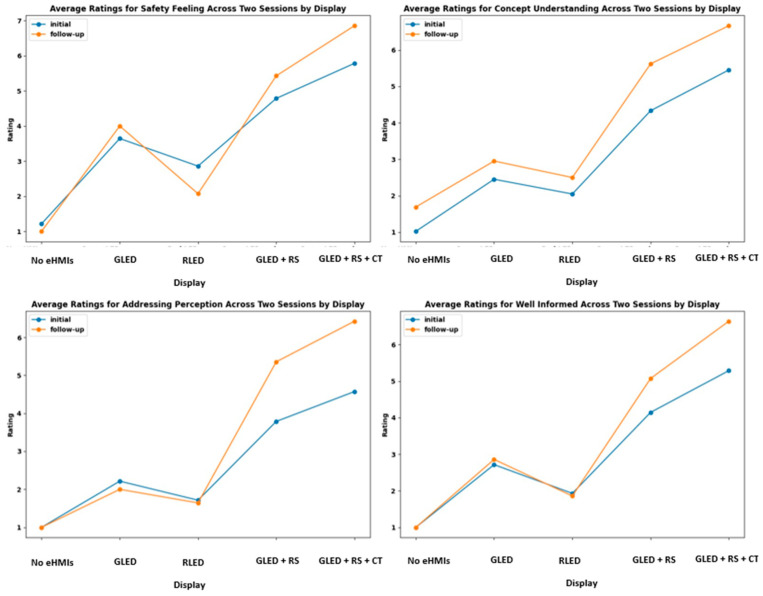
Comparison of the participants’ mean ratings for the initial and the follow-up sessions.

Figure 11 reveals that there is a marked improvement in the participants’ ratings for the eHMI displays during the follow-up session. This improvement was particularly noticeable for a display such as the “green LED combined with robotic sign and timer”. The absence of eHMIs consistently registered lukewarm responses, emphasizing the essential role of eHMIs in fostering pedestrian trust. When delving into the nuances of concept understandability and the perception of being addressed, it became evident that while certain configurations, like the “green LED combined with robotic sign”, began on a modest note during the initial session, their efficacy saw a commendable surge during the follow-up, suggesting an evolving resonance or perhaps heightened clarity over time. 

Statistical analyses, specifically paired-sample *t*-tests, were conducted to compare the participant ratings between the initial and follow-up sessions. This test is appropriate because we are comparing two sets of related data (initial and follow-up) collected from the same group of participants. For the “safety feeling” metric, no statistically significant difference was observed between the initial and follow-up sessions (t = −1.31, *p* = 0.20), suggesting stability in the participants’ feelings of safety across the study duration. In contrast, in the participants’ understanding of the concept, as gauged by the “concept understanding” metric, a statistically significant difference was identified between the two sessions (t = −7.27, *p* < 0.001). This underscores a statistically significant enhancement in concept comprehension between the initial and follow-up evaluations. Similarly, both the “addressing perception” and “well informed” metrics showed significant differences, with t-values of −3.86 and −3.68, and *p*-values of <0.001 and <0.001, respectively. These results indicate that the participants’ feelings of being addressed and being well informed evolved between the two sessions. 

### 4.3. Intercept Survey Results

#### 4.3.1. Demographics Results 

The age distribution of the participants was varied, with the largest group being those aged 18–24 years, comprising 37% of the total. This was followed by the 25–40 age group, representing 32%, and the 41–64 age group, accounting for 24%. The participants over 64 years of age made up 7% of the total, with 12 individuals in this category. Regarding gender distribution, males formed the majority, constituting 56%, while females accounted for 41%. Additionally, a small number of participants, 3%, did not specify their gender. 

#### 4.3.2. Willingness to Cross 

**Figure 12:**
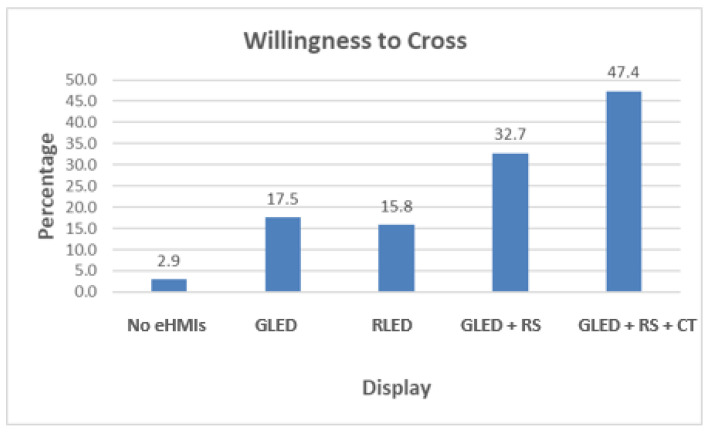
Percentage of participants who are willing to cross per display.

The participants’ willingness to cross in the presence of different eHMI displays is illustrated in (Figure 12). Among the 171 respondents, only a small fraction, 3%, expressed their willingness to cross in scenarios where no eHMIs were present. This indicates a general reluctance to interact with AVs without any communication cues. When a flashing green LED light was used as the eHMI, the willingness to cross increased, with 17.5% (30 participants) feeling comfortable enough to proceed. The flashing red LED light saw a similar level of confidence, with 15.8% (27 participants) indicating their willingness to cross. The combination of a flashing green LED with a robotic sign marked a notable increase in the participants’ willingness to cross, jumping to 32.7% (56 participants). This suggests that the added element of the robotic sign enhanced the participants’ understanding and trust in the vehicle’s intent. The highest willingness to cross was observed when the eHMI display combined a flashing green LED, a robotic sign, and a countdown timer, with 47.4% (81 participants) indicating readiness to cross. This combination appeared to provide the most clarity and assurance to the participants, highlighting the effectiveness of a more comprehensive eHMI system in communicating the vehicle’s intentions in pedestrian interactions.

#### 4.3.3. Concept Understanding 

**Figure 13:**
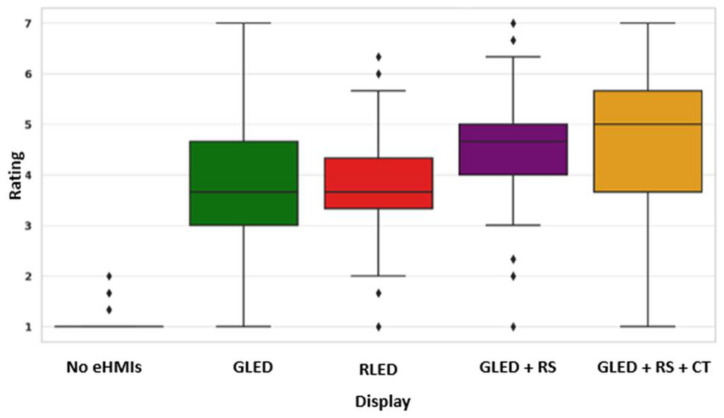
Participants’ rating for concept understanding across displays.

The boxplot below (Figure 13) represents the participant concept understanding ratings for the various eHMI displays. The displays received distinct ratings from the participants, indicating varying levels of understandability. The “no eHMIs” option, with a median rating of 1.0 and several outliers, serves as a baseline, indicating that any eHMI is generally preferred over no interface. The “green LED” display showed no outliers and a moderate IQR, indicating a relatively consistent perception among the participants. The “red LED” display had a number of outliers, indicating varied opinions and interpretations among the participants. The “green LED + robotic sign” and “green LED + robotic sign + timer” displays have the highest median ratings, suggesting they are the most understandable among the options. The display with the highest median rating emerges as the most understandable by pedestrians, suggesting it aligns best with their expectations and is the easiest for them to interpret. This clarity in communication is crucial for pedestrian safety and effective interaction with AVs.

The results of our study indicate a clear preference for certain eHMI configurations among pedestrians. In particular, the combination of a green LED, robotic sign, and timer was consistently preferred across different methods of data collection (see Table 3). This preference was evident in increased pedestrian crossing willingness, faster response times, and higher perceived safety ratings. The participants’ preference for this combination can be attributed to several factors. The green LED provided a clear and visible signal that was easily distinguishable from other traffic elements, likely contributing to the high levels of perceived safety and confidence in crossing decisions. The inclusion of a timer offered dynamic, real-time information about the remaining time to cross, reducing uncertainty and improving the participants’ decision-making processes, which led to quicker response times. The robotic sign, designed to replace the driver and indicate that the vehicle was in automated driving mode, added an element of intuitive design. Although the robotic sign did not involve gestures, its clear and recognizable symbol effectively communicated the AV’s operational state to the pedestrians, enhancing overall comprehension and trust in the eHMI. Additionally, the use of multiple signals (visual and dynamic) reinforced the AV’s intentions, reducing ambiguity and increasing the pedestrians’ willingness to cross. The combination of these elements provided a robust and reliable means of communication that the participants found both informative and reassuring.

## 5. Discussion

The integrated analysis of our experiments and the survey study provides a nuanced understanding of pedestrian interactions with AVs facilitated by eHMIs. Consistent with the existing literature, our research supports the principle that clear signaling via eHMIs is crucial for improving pedestrian safety and crossing decisions. This is evident in the observed trends across our studies, where the eHMI displays that provide clear information—such as the green LED with robotic sign and countdown timer—significantly increased the pedestrians’ willingness to cross, crossing response time, as well as their perception and understanding.

The survey findings of our study, which sampled a broader demographic, confirmed the experimental findings. A significant majority of the survey participants expressed higher confidence in crossing with the green LED combined with a robotic sign and timer compared to the other eHMI configurations. This preference was also observed in our experiments, demonstrating a 100% willingness to cross in the follow-up experiment, confirming a general preference for more informative eHMI displays. Our findings align with those of [18], suggesting that pedestrians want to know about a vehicle’s driving status and what it plans to do.

Concept understanding ratings closely mirrored the trends in crossing willingness, suggesting that as pedestrians understand the eHMI’s message better, they feel more confident in making crossing decisions. However, the survey study revealed a surprising aspect of participant behavior concerning red and green LED displays. Both interfaces received nearly equivalent willingness to cross percentage and concept understanding ratings, suggesting that the color of the LED alone may not be a primary factor in pedestrian comprehension. These findings are consistent with the results of the initial experiment where some participants mentioned that interpreting color is challenging as well as they are in line with the results from the [36] study. This outcome points towards a need for more research into how pedestrians interpret these cues in real-world scenarios and the potential for innovative, perhaps non-color-reliant, eHMI solutions.

Our study highlights the effectiveness of specific eHMI configurations; however, implementing these eHMI designs in diverse urban environments presents several potential challenges. Urban environments vary significantly in terms of infrastructure, traffic density, and pedestrian behavior. Implementing eHMIs that are effective across such varied contexts requires careful consideration of local conditions and the potential customization of eHMI elements. Additionally, integrating eHMIs into the existing AV systems involves technical challenges, such as ensuring compatibility with various vehicle models and maintaining reliable operation under different environmental conditions (e.g., weather and lighting). Moreover, achieving the widespread public acceptance of new eHMI designs requires extensive public education and outreach. Pedestrians need to be familiarized with the signals and their meanings to ensure effective communication. Furthermore, the adoption of eHMIs on a large scale will require regulatory approval and standardization across different regions and countries. Establishing common standards for eHMI design and implementation is crucial for consistency and effectiveness.

While our study provides valuable insights, it is not without limitations. The sample size, though adequate, may not fully represent the diversity of urban populations. Additionally, the profile of the participants may pose a limitation. Most participants were young adults, which may not fully represent the diversity of the general population. Future studies should aim to include a broader demographic to enhance the generalizability of the findings. Moreover, the controlled nature of the experimental setup and the used scenarios might not encompass all real-world traffic scenarios. Acknowledging these limitations is crucial in understanding the scope and applicability of our findings. Future research could explore the impact of cultural and demographic factors on eHMI interpretation and effectiveness. Additionally, long-term studies observing pedestrian behavior over extended periods could provide deeper insights into how familiarity with eHMIs influences pedestrian behavior.

While our study primarily focused on pedestrian–AV interactions, the presence of other traffic participants, such as cyclists, can significantly influence pedestrian behavior. Interactions with cyclists may add to the complexity of the traffic environment, potentially affecting pedestrians’ decision-making processes and crossing behaviors. For instance, the presence of cyclists might cause pedestrians to hesitate or alter their crossing path to avoid potential collisions. Future research should investigate these dynamics to provide a more comprehensive understanding of mixed-traffic interactions and the effectiveness of eHMIs in such environments.

## 6. Conclusions

In summary, our study thoroughly investigated the impacts of various eHMI displays on pedestrian interaction with AVs. The experiments and accompanying survey study revealed that certain eHMI displays, particularly the combination of a green LED, robotic sign, and timer, significantly enhanced pedestrian crossing willingness, response time, safety feeling, and concept clarity. It can be concluded that this concept promises to provide a scalable way of communication. It can be concluded that pedestrians’ safe communication with AVs lies in receiving sufficient and required information such as the AV’s driving mode, intention, and the time available for pedestrians to cross.

The practical applications of our research are significant. The identified eHMI configurations provide a clear direction for designing interfaces that enhance pedestrian safety and confidence in AV interactions. City planners and designers can use these findings to develop safer pedestrian crossings and integrate eHMIs into urban infrastructure, thereby improving overall traffic safety. The manufacturers of AVs can incorporate the recommended eHMI designs into their vehicles, ensuring that AVs communicate effectively with pedestrians, thereby reducing the likelihood of accidents and enhancing public trust in AV technology. Policymakers can use the insights from this study to develop guidelines and regulations for eHMI implementation, ensuring that the benefits of eHMIs are realized across different urban settings.

Our findings provide a foundation for future research on eHMIs in urban environments. By demonstrating the effectiveness of specific eHMI configurations in real-world scenarios, this study paves the way for further exploration into scalable eHMI designs. Future research can build on our work by investigating the long-term impact of eHMI exposure on pedestrian behavior and expanding the scope to include diverse traffic participants and environments. This will contribute to the development of more intuitive and universally applicable eHMIs, ultimately enhancing traffic safety and efficiency.

Our study significantly contributes to the understanding of pedestrian–AV interactions, demonstrating the critical role of eHMI design in enhancing pedestrian safety and confidence. The consistent preference for the combined green LED, robotic sign, and timer display across the different methods of data collection underscores the robustness of these findings and their significance for future AV deployments and the advancement of traffic safety standards.

## Figures and Tables

**Table 1:** Mixed-effects model results for participant crossing response time.

Variable	Coef.	Std. Err.	z	*p* > |z|
Intercept	12.329	1.685	7.317	0.000
Display [T.Green LED]	−3.982	1.814	−2.195	0.028
Display [T.Green LED and Robotic sign]	−4.865	1.777	−2.738	0.006
Display [T.Green LED and Robotic sign and Timer]	−5.465	1.723	−3.171	0.002
Display [T.Red LED]	−2.156	1.962	−1.099	0.0272
Scenario	1.354	1.040	1.302	0.193
Display [T.Green LED]: Scenario	−0.079	1.164	−0.068	0.946
Display [T.Green LED and Robotic sign]: Scenario	0.318	1.119	0.285	0.776
Display [T.Green LED and Robotic sign and Timer]: Scenario	−0.132	1.083	−0.122	0.903
Display [T.Red LED]: Scenario	0.379	1.240	0.306	0.760
Group Var	2.204	0.874		

**Table 2:** Comparison of pedestrian willingness to cross per display and session.

Display	Willingness to Cross (%)	Willingness to Cross (%)
	Initial	Follow-Up
Green LED and Robotic sign and Timer	71.43%	100.00%
Green LED and Robotic sign	28.57%	57.14%
Green LED	21.43%	28.57%
Red LED	7.14%	0.00%
No eHMIs	0.00%	0.00%

**Table 3:** Comparative impact of eHMIs on pedestrians in mixed-traffic situations across studies.

Display/Study	Initial Experiment (31 Participants)	Follow-Up Experiment (14 Participants)	Survey (171 Participants)
	Willingness to Cross (%)		
No eHMI	6	0	3
Green LED	19	29	18
Red LED	13	0	16
Green LED + Robotic Sign	32	57	33
Green LED + Robotic Sign + Countdown Timer	65	100	47
	Concept Understanding Rating (mean)		
No eHMI	1.0	1.7	1.0
Green LED	2.5	3.0	3.8
Red LED	2.0	2.5	3.8
Green LED + Robotic Sign	4.3	5.6	4.7
Green LED + Robotic Sign + Countdown Timer	5.5	6.7	5.0
	Safety Feeling Rating (mean)		
No eHMI	1.2	1.0	N/A
Green LED	3.6	4.0	N/A
Red LED	2.9	2.1	N/A
Green LED + Robotic Sign	4.8	5.4	N/A
Green LED + Robotic Sign + Countdown Timer	5.8	6.9	N/A
	Feeling Addressed Rating (mean)		
No eHMI	1.0	1.0	N/A
Green LED	2.2	2.0	N/A
Red LED	1.7	1.6	N/A
Green LED + Robotic Sign	3.8	5.4	N/A
Green LED + Robotic Sign + Countdown Timer	4.6	6.4	N/A
	Feeling Well Informed Rating (mean)		
No eHMI	1.0	1.0	N/A
Green LED	2.7	2.9	N/A
Red LED	1.9	1.9	N/A
Green LED + Robotic Sign	4.1	5.1	N/A
Green LED + Robotic Sign + Countdown Timer	5.3	6.6	N/A
	Crossing Response Time (mean in second)		
No eHMI	15.55	N/A	N/A
Green LED	11.62	9.78	N/A
Red LED	13.58	N/A	N/A
Green LED + Robotic Sign	10.35	9.01	N/A
Green LED + Robotic Sign + Countdown Timer	9.06	7.94	N/A

## Data Availability

The data presented in this study are available on request from the corresponding author due to ongoing research.
